# Process evaluation of an implementation trial to improve the triage, treatment and transfer of stroke patients in emergency departments (T^3^ trial): a qualitative study

**DOI:** 10.1186/s13012-020-01057-0

**Published:** 2020-11-04

**Authors:** Elizabeth McInnes, Simeon Dale, Louise Craig, Rosemary Phillips, Oyebola Fasugba, Verena Schadewaldt, N. Wah Cheung, Dominique A. Cadilhac, Jeremy M. Grimshaw, Chris Levi, Julie Considine, Patrick McElduff, Richard Gerraty, Mark Fitzgerald, Jeanette Ward, Catherine D’Este, Sandy Middleton

**Affiliations:** 1 Nursing Research Institute - St Vincent’s Health Network Sydney, St Vincent’s Hospital Melbourne & Australian Catholic University, School of Nursing, Midwifery & Paramedicine, Australian Catholic University, Level 4, Daniel Mannix Building, Brunswick Street, Fitzroy, Victoria 3065 Australia; 2 Nursing Research Institute - St Vincent’s Health Network Sydney, St Vincent’s Hospital Melbourne & Australian Catholic University, Level 5, deLacy Building, Victoria Street, Darlinghurst, New South Wales 2010 Australia; 3 Nursing Research Institute - St Vincent’s Health Network Sydney, St Vincent’s Hospital Melbourne & Australian Catholic University, Level 2, Signadou Building, Australian Catholic University, 223 Antill Street, Watson, Australian Capital Territory 2602 Australia; 4 Department of Neurosurgery, University of Melbourne and Royal Melbourne Hospital, Grattan Street, Parkville, Victoria 3050 Australia; 5 Formerly: Nursing Research Institute - St Vincent’s Health Network Sydney, St Vincent’s Hospital Melbourne & Australian Catholic University, Victoria, Australia; 6 Centre for Diabetes and Endocrinology Research, Westmead Hospital and University of Sydney, Westmead, Sydney, New South Wales Australia; 7 Stroke and Ageing Research, School of Clinical Sciences at Monash Health, Monash University, Clayton, Victoria Australia; 8 Florey Institute of Neuroscience and Mental Health, University of Melbourne, Parkville, Victoria Australia; 9 Clinical Epidemiology Program, Ottawa Health Research Institute, Ottawa Hospital – General Campus, Centre for Practice-Changing Research (CPCR), 501 Smyth Road, Room 1286, Ottawa, Ontario K1H 8 L6 Canada; 10 Department of Medicine, University of Ottawa, 451 Smyth Road, Ottawa, Ontario K1H 8 M5 Canada; 11 The Sydney Partnership for Health Education Research & Enterprise (SPHERE), University of New South Wales, Liverpool, New South Wales Australia; 12 School of Nursing and Midwifery, Deakin University, Geelong, Victoria Australia; 13 Centre for Quality and Patient Safety Research, Institute for Health Transformation, Deakin University, Geelong, Victoria 3220 Australia; 14 School of Medicine and Public Health, University of Newcastle, Newcastle, New South Wales Australia; 15 Department of Medicine, Monash University, Melbourne, Victoria 3800 Australia; 16 Neurosciences Clinical Institute, Epworth Hospital, Richmond, Victoria 3121 Australia; 17 Department of Surgery, Central Clinical School, Monash University, Melbourne, Victoria 3800 Australia; 18 Faculty of Science, Engineering and Technology, Swinburne University of Technology, Melbourne, Australia; 19 Nulungu Research Institute, University of Notre Dame Australia, Broome, Western Australia Australia; 20 National Centre for Epidemiology and Population Health (NCEPH), Australian National University, Canberra, Australian Capital Territory 0200 Australia; 21 School of Medicine and Public Health, The University of Newcastle, Callaghan, New South Wales 2308 Australia

**Keywords:** Process evaluation, Normalisation process theory, Acute stroke, Emergency departments, Qualitative design

## Abstract

**Background:**

The implementation of evidence-based protocols for stroke management in the emergency department (ED) for the appropriate triage, administration of tissue plasminogen activator to eligible patients, management of fever, hyperglycaemia and swallowing, and prompt transfer to a stroke unit were evaluated in an Australian cluster-randomised trial (T^3^ trial) conducted at 26 emergency departments. There was no reduction in 90-day death or dependency nor improved processes of ED care. We conducted an a priori planned process influential factors that impacted upon protocol uptake.

**Methods:**

Qualitative face-to-face interviews were conducted with purposively selected ED and stroke clinicians from two high- and two low-performing intervention sites about their views on factors that influenced protocol uptake. All Trial State Co-ordinators (*n* = 3) who supported the implementation at the 13 intervention sites were also interviewed. Data were analysed thematically using normalisation process theory as a sensitising framework to understand key findings, and compared and contrasted between interviewee groups.

**Results:**

Twenty-five ED and stroke clinicians, and three Trial State Co-ordinators were interviewed. Three major themes represented key influences on evidence uptake: (i) *Readiness to change:* reflected strategies to mobilise and engage clinical teams to foster cognitive participation and collective action; (ii) *Fidelity to the protocols*: reflected that beliefs about the evidence underpinning the protocols impeded the development of a shared understanding about the applicability of the protocols in the ED context (coherence); and (iii) *Boundaries of care:* reflected that appraisal (reflexive monitoring) by ED and stroke teams about their respective boundaries of clinical practice impeded uptake of the protocols.

**Conclusions:**

Despite initial high ‘buy-in’ from clinicians, a theoretically informed and comprehensive implementation strategy was unable to overcome system and clinician level barriers. Initiatives to drive change and integrate protocols rested largely with senior nurses who had to overcome contextual factors that fell outside their control, including low medical engagement, beliefs about the supporting evidence and perceptions of professional boundaries. To maximise uptake of evidence and adherence to intervention fidelity in complex clinical settings such as ED cost-effective strategies are needed to overcome these barriers.

**Trial registration:**

Australian New Zealand Clinical Trials Registry (ACTRN12614000939695).

Contributions to the literature

- Process evaluations are important to conduct alongside complex implementation trials. Their value is particularly evident when a multicomponent, evidence-based intervention informed by a theoretical behaviour change framework did not change clinician behaviour or patient outcomes.
- This a priori planned process evaluation, informed by the Normalisation Process Theory, identified complex individual and contextual factors that hindered uptake of evidence based protocols for acute stroke that could not be overcome in emergency departments (ED).
- These findings contribute to the body of knowledge about the implementation of research evidence into clinical practice in the complex ED setting and will help guide the implementation of future interventions for acute stroke care in the ED.

## Background

Worldwide, stroke is a major cause of mortality and disability [1]. In 2016, there were 13.7 million new stroke cases and 5.5 million deaths from stroke and incidence of stroke are increasing in some countries including east Asia and sub-Saharan Africa [1]. Costs associated with stroke care are substantial. A population-based cost analysis of 32 European countries estimated 60 billion euros, with health care accounting for 27 billion euros (45%) [2]. In Australia, expenditure related to stroke is estimated to cost the economy around $5 billion per annum [3].

Evidence-based stroke care can improve survival following stroke [4]. National clinical guidelines include recommendations for rapid clinical assessment, early diagnosis, and evidence-based management of patients with stroke in the emergency department (ED) [5]. Essential protocol elements include appropriate triage (categories 1 or 2 are recommended in Australia for patients with neurological deterioration) [6]; administration of tissue plasminogen activator (thrombolysis) for eligible patients; prompt transfer to an acute stroke unit; and, as shown in our earlier trial, management of fever, hyperglycaemia and swallowing [4, 5]. Given that there are demonstrated health and economic benefits from implementing evidence-based stroke protocols with an estimated cost saving of $281 million a year, delivering evidence-based care in the ED could have the potential to further improve patient outcomes nationally and internationally [7, 8].

Delivering optimal evidence-based care to patients with stroke in EDs, whilst managing other patients with a broad range of illnesses and injuries of varying severity and clinical urgency is challenging [8, 9]. Hence, providing EDs with support to deliver evidence-based triage, treatment and transfer of patients presenting with acute stroke has the potential to improve multidisciplinary care and patient outcomes.

A prospective, multicentre, parallel-group and cluster-randomised controlled trial (C-RCT) was undertaken to evaluate the effect of a multicomponent evidence-based intervention to improve the Triage, Treatment and Transfer (T^3^) and 90-day health outcomes of acute stroke patients in 26 Australian EDs across three states and one territory [10]. The intervention (Table 1) was based on one that previously had been successfully used in stroke units (QASC trial) [4] and consisted of multidisciplinary supported, nurse-led evidence-based protocols and an implementation strategy informed by behavioural change theory and the Theoretical Domains Framework [11–13]. While there were a priori minimum care elements and a fixed evidence-based implementation strategy, flexible local implementation adaptation was permitted, and regular proactive and reactive implementation support was given to the two nursing clinical champions at each intervention site (one a stroke senior nurse and one a senior emergency nurse) by Trial State Co-ordinators. The ED Directors at each intervention site agreed to support implementation. While the intervention was delivered as planned at the cluster level, there was no statistically significant difference between patients in the intervention and control groups for 90-day death or dependency and no improvements in ED clinician behaviour for key ED stroke care practices [10]

**Table 1:** T^3^ Trial intervention components

**T**^**3**^ **clinical protocols**	
***Triage***	
• All patients presenting with signs and symptoms of suspected stroke should be triaged to Australasia Triage Scale (ATS) categories 1 or 2 (seen within 10 min)	
***Treatment***	
Thrombolysis (tissue-type plasminogen activator)	
• All patients to be assessed for thrombolysis eligibility	
• All eligible patients to receive thrombolysis	
Fever	
• All patients to have their temperature taken on admission to emergency department (ED) and then at least four hourly whilst they remain in ED	
• Treat temperature 37.5 °C or greater with paracetamol within 1 h	
Sugar	
• Formal venous (laboratory) blood glucose level (BGL) on admission to ED	
• Record finger prick BGL on ED admission and monitor finger prick BGL every 6 h (or greater if elevated)	
• Administer insulin to all patients with BGL > 10 mmol/L (180 mg/dL) within 1 h	
Swallow	
• Patients remain Nil By Mouth until a swallow screen by non-speech pathologist (SP) or swallow assessment by SP performed, i.e.:	
◦ No oral food or fluids to be given prior to swallow screen by non-SP or swallow assessment by SP	
◦ No oral medications administered prior to swallow screen by non-SP or swallow assessment by SP	
• All patients who fail the screen are to be assessed by a SP	
***Transfer***	
• All patients with stroke to be discharged from ED within 4 h	
• All patients with stroke to be admitted to the hospital’s stroke unit	
**T**^**3**^ **Implementation strategy**	
***Multidisciplinary Workshops***^a^	
*Workshop 1 - Barriers and Enablers Assessment (one at each site, 60 min)*	
• To present the details of the trial	
• To identify the local barriers and enablers	
• To identify the local site clinical champion	
*Workshop 2 - Action Plan (one at each site, 60 min)*	
• To discuss the action plan to address the barriers	
• To ascertain the actions already taken to address the barriers	
• To identify the new local barriers	
***Didactic and interactive education***^a^ *(minimum one at each site, 30 min)*	
• A 20-min PowerPoint presentation and a 10-min discussion	
• An 8-min video developed by an academic ED nurse clinician/opinion leader	
***Use of clinical opinion leaders***	
• Key national clinical opinion leaders at Workshop 1 and available as needed for any site-requested queries	
• Clinical champions from ED and stroke unit	
• ***Reminders***	
• Reminder poster to display in ED- and pocket-sized card to attach to ID lanyard for staff	
• Proactive direct contact every 6 weeks in the form of the following:	
◦ Site visits every 3 months (face-to-face) using an action plan	
◦ Teleconferences every 3 months with clinical champions and site coordinator using an action plan	
• Emails—reactive and monthly proactive emails	
• Telephone support—reactive	
• Telephone support—reactive and as needed	

“On admission” defined as within 60 min of arrival to ED. Four hourly defined as within 4.5 h. Six hourly defined as within 6.5 h

^a^Face-to-face multidisciplinary group sessions held at each intervention site

Process evaluations are conducted following a pragmatic implementation trial such as the T^3^ Trial and seek to understand the factors that influence intervention uptake [14]. Normalisation process theory (NPT) can be used to understand processes associated with the implementation, embedding and integration of new practices in complex and dynamic health settings [15, 16]. The NPT concepts of coherence (does the new practice make sense?), cognitive participation (how engaged are individuals with practice change), collection action (how were the new practices integrated and use facilitated) and reflexive monitoring (value of and impact of the new practices on the team) focus on individual collective and contextual influences and are useful for exploring and understanding protocol uptake in complex settings [15, 16]. As we had planned a priori to undertake a process evaluation at the end of the trial, the NPT was considered to provide a useful sensitising framework for understanding what facilitated or hindered implementation uptake of the T3 protocols. The aim of this qualitative process evaluation was to understand from the viewpoint of those involved in implementation, the factors that influenced the adoption of evidence-based protocols in the management of acute stroke presentation in the ED and to identify barriers and facilitators to protocol implementation and uptake.

## Methods

**Fig. 1:**
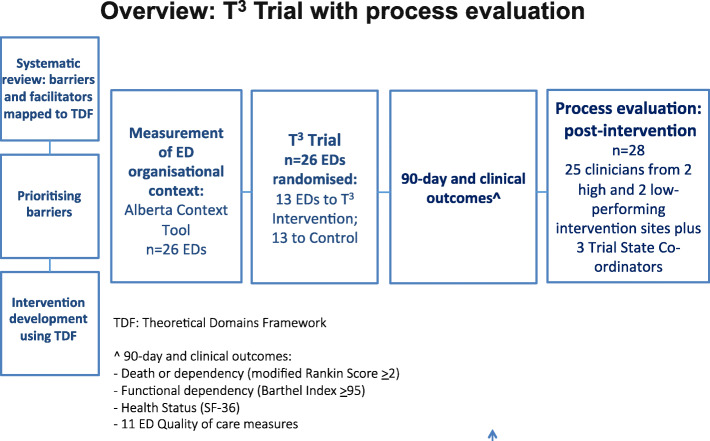
Overview of T^3^ Trial with process evaluation

This qualitative process evaluation was designed prior to commencement of the C-RCT and conducted from mid-August to late September 2017 following the conclusion of the T^3^ Trial which is detailed elsewhere [10] (see Fig. 1). Frameworks for the design and reporting of the process evaluation of complex interventions developed by the United Kingdom (UK) Medical Research Council [14] and C-RCTs [17] informed the design, conduct and reporting of this study. A qualitative descriptive design [18] was used to interview two groups of participants: ED and stroke clinicians involved in the implementation of the T^3^ clinical protocols and the Trial State Co-ordinators who were responsible for supporting the intervention sites. Qualitative descriptive design was chosen as it enables a rich description of first-hand experiences or an event that is grounded in the participants’ words. Although the Theoretical Domains Framework was useful for characterising potential barriers to the implementation of the T3 protocols and for informing the development of the intervention strategy [11–13], the normalisation process theory (NPT) informed the interview guide and analysis and was chosen because it emphasises the role of individual interactions and collective working in implementation processes.

### Setting

The T^3^ Trial enrolled 26 hospitals in three Australian states (New South Wales, Victoria, Queensland) and the Australian Capital Territory that had a 24-h emergency department and dedicated stroke units and randomly allocated 13 EDs to the intervention group and 13 to the control group.

### Sampling

#### Hospitals for process evaluation

For each of the 13 intervention sites, processes of care data for the 13 components of the T^3^ clinical protocols were obtained from the medical record of each patient trial participant [11] and a score out of 13 allocated for the number of processes of care correctly adhered to. An average score for each of the 13 EDs was calculated and then EDs were ranked from highest to lowest performing and divided into quartiles. Two EDs were randomly selected by an independent statistician from the top and bottom quartiles where the T^3^ clinical protocol uptake was ‘good’ (two high-performing sites) and ‘poor’ (two low-performing sites).

#### Participants for process evaluation interviews

From the four intervention hospitals classified as high- and low-performing as described above, purposive maximum variation sampling was used to select participants amongst different disciplines for the interview (Table 2). Participants had to have been either engaged in the direct care of patients with stroke during the T^3^ Trial and/or had local oversight of the implementation of the T^3^ clinical protocols. The sample included more nurses as the protocols were designed to be nurse-led with two nurse clinical champions at each site, hence the sample included more nurses. ED Medical Directors, as leaders had a key role in endorsing and influencing the use of the protocols. The three Trial State Co-ordinators, who all had a health background (nursing or psychology), were also approached to be interviewed. Participants provided written consent.

**Table 2:** Description of participants by group

Participant group	Description
ED medical director	Partner researcher and senior medical officer responsible for delivery of ED medical services and for supporting implementation
ED nurse unit manager	Emergency department nurse manager with detailed knowledge of ED clinical operation.
ED clinical champion	Senior emergency nurse clinician/educator from participating hospital’s ED appointed as clinical champion to support intervention implementation and liaise with the Trial State Co-ordinator
Stroke unit clinical champion	Senior nurse clinician or educator from participating hospital’s stroke unit appointed as clinical champion to support intervention implementation and liaise with ED clinical champion
ED nurse	Nurses involved in delivery of emergency care
Trial state co-ordinator	Nurse/psychology graduate employed to provide support to intervention sites to address barriers (one co-ordinator each for New South Wales/Australian Capital Territory, Victoria and Queensland)

### Data collection

The semi-structured interview guide was designed to facilitate exploration of participants’ views of factors that influenced the introduction, implementation and embedding of the T^3^ clinical protocols into practice. This comprised open-ended questions about the participant’s role in implementation; their views on whether clinical teams understood what was required of them in relation to implementation and protocol use; their views on barriers and facilitators to protocol uptake including organisational, workplace and individual factors and views on sustainability. NPT concepts [15, 16] informed the interview guide as follows: whether the aims and benefits of the T3 protocols were understood (*coherence*); actions implemented to facilitate staff awareness and engagement in protocol use (*cognitive participation*); how protocol uptake was facilitated (*collective action*); and whether and how the protocols impacted upon roles, responsibilities and care delivery (*reflexive monitoring*) [15, 16]. Interview schedules for the hospital clinicians and Trial State Co-ordinators contained a core set of similar questions plus additional questions that reflected their different roles. The interview schedule was pilot-tested for meaningfulness and clarity in two mock interviews and minor revisions were made.

Interviews were conducted 12 months after cessation of the intervention. Interviewers were independent from the study investigator team and whilst not blinded to site classification (low- or high-performing site) were unaware of each site’s individual performance data and were blinded to the trial results. Emergency nurses participated in group interviews at each site as it was difficult to schedule individual interviews because of the unpredictable nature of bedside clinical work. Participants were assured of the confidentiality of their and their organisations’ identity through the use of pseudonyms. All interviews were audio-recorded, anonymised and professionally transcribed.

### Analysis

Demographic and professional data were summarised using means and counts as appropriate. Analysis of narrative interview data involved three stages. The interview framework was transposed into a matrix that was used to manage data from each of the four research sites. Data were imported into the matrices and partitioned by a professional group, then organised and coded. First, deductive content analysis with open coding was performed guided by, but not limited to, NPT concepts which provided a sensitizing framework for key findings. The second stage, code reduction, was achieved by convergence of codes that related to similar data and was conducted both within and across professional groups and then research sites. All data matrices were cross-checked to identify the stability of codes within the interview framework and across professional groups and research sites [19]. A third analytical step identified a set of themes and sub-themes. To minimise researcher bias, the researchers initially coded transcripts independently and then met to discuss and compare codes and themes. Discrepancies were discussed and resolved. When needed, the researchers consulted the transcripts to ensure consistent interpretation and also linked participant quotes to the themes to illustrate clear links between the agreed themes and the data. The data were judged as saturated when no new issues or insights between sites or between participant groups were reported.

### Ethics

Approval was obtained from the Human Ethics Research Committees (HRECs) from the Australian Catholic University (2012 16 N), Sydney Local Health District (Royal Prince Alfred Hospital Zone) (HREC/12/RPAH/32) (NSW Lead Ethics Committee) and other multiple sites.

## Results

### Characteristics of participants

A total of twenty-eight interviews were completed; 25 clinicians and 3 Trial State Co-ordinators (Table 3). Ten emergency nurses were interviewed in groups comprising of 2–3 participants per session. The duration of the interviews was between 30 and 45 min. Overall, the responses were highly consistent across the four sites and participant groups.

**Table 3:** Demographic and professional characteristics of participants (*n* = 28)

Participant group	Median number of years in ED or stroke care (range)	Sex ratio female/male
ED medical director (*n* = 4)	18.5 (15–20)	All males
ED nurse unit manager (*n* = 3)	18.0 (16–20)	All females
ED clinical champion (*n* = 4)	13.5 (8–20)	3:1
Stroke unit clinical champion (*n* = 4)	9.0 (4–13)	3:1
ED nurse (*n* = 10)	6.8 (2.5–12)	All females
Trial state co-ordinator (*n* = 3)	N/A	All females

Three main themes with sub-themes were identified that captured the major factors to the uptake of the T^3^ clinical protocols and were linked to NPT concepts (Table 4).

**Table 4:** Themes and sub-themes

Themes and sub-themes	Normalisation process theory concept
**Readiness to change** Mobilising teams Authority to implement	**Cognitive participation and collective action:** Perceived authority of nurse clinical champions to drive change resulted in challenges in achieving collective engagement.
**Fidelity to protocols** Engaging with the evidence Adherence versus adaptation	**Coherence:** Beliefs about the evidence underpinning the protocols impeded the development of a shared understanding about the applicability of aspects of the protocols in the ED
**Boundaries of care** Defining professional boundaries Care trajectory	**Reflexive monitoring:** The extent to which the protocols were implemented was influenced by perceptions of clinical roles and responsibilities throughout the patient trajectory.

### Readiness to change

#### Mobilising teams

In terms of the benefits of participating in the trial, most participants cited that improved patient outcomes were a motivating factor for practice change. Emergency department nurses and clinical champions, in particular, articulated valuing the opportunity to ‘make a difference’ to patient outcomes. The importance of clinical champions in leading implementation and facilitating staff across all shifts and across the ED and Stroke teams, to ‘pull together’ was emphasized.

[The] clinical champion needs time and ability to drive the protocols daily in ED. Needs to be really tenacious and live and breathe the protocols (Stroke Unit Senior Nurse)

It’s been really good building up relationships with the stroke team because often you get caught in your little silos (Senior Emergency Nurse)

The extent of staff movement and the volume of staff in the ED meant that frequently clinical champions had to *Educate clinicians at the point of care with a patient in front of them* (Senior Emergency Nurse) to ensure that good coverage of education about the protocols across staff groups. However, clinical champions reported challenges in relation to engaging medical staff and some participants expressed the view that a medical clinical champion was needed as well to help drive change across the multidisciplinary team and to support education about the protocols:

Medical head of department needed to stamp authority, otherwise you struggle to have any impact (Stroke Unit Senior Nurse)

(You) need also medical champions – not just nurse champions (Trial State Co-ordinator)

The lack of medical support resulted in clinical champions feeling solely responsible for implementation, staff education and for mobilising collective interdisciplinary momentum for practice change.

#### Authority to implement

The T^3^ clinical protocols were multidisciplinary but designed to be nurse-led. The clinical champions at each hospital were required to drive implementation and to ensure that clinical practice aligned with the protocols. Participants (nursing and medical) stated that the importance of the authority of nursing to implement the protocol and drive practice change was central to whether protocol uptake occurred. While some participants reported that the protocols gave authority to clinical champions when negotiating collaboration over aspects of stroke patient care, other participants felt that clinical champions needed to be ‘more authoritative when implementing evidence’ (Stroke Unit Senior Nurse). Trial State Co-ordinators expressed the belief that clinical champions varied in their ability to be influential, to obtain ongoing buy-in from colleagues and to navigate clinical boundaries and build inter-disciplinary relationships:

It all relates back to team dynamics and also the type of leaders and how people are respected and listened to within the team. I felt that sometimes (a clinical champion) didn’t get the respect and support from the medical lead. It was like the clinical champion was a little bit powerless (Trial State co-ordinator)

Promoting the multidisciplinary protocols as nurse-led was also perceived to hamper a shared understanding of the protocols as multidisciplinary:

… if you’re going to refer to nurse-led changes, then doctors would instantly assume, that’s for nurses, I don’t need to be doing that. I feel like that feeds the silo approach and it would be better to say interdisciplinary protocols (Emergency nurse)

In summary, achieving multidisciplinary education and medical engagement was challenging for clinical champions who were perceived to ‘lack authority’ or have insufficient authority to ensure collective investment in the implementation of the protocols.

### Fidelity to protocols

#### Engaging with the evidence

Participant feedback reflected variable engagement with the research evidence underpinning the protocols, despite the supporting evidence and rationale being presented at multidisciplinary workshops and at individual meetings with stroke, ED and endocrinology medical staff. A number of participants reported that they or others in the ED team expressed beliefs at odds with aspects of the evidence on which the protocols (and supporting national and international guidelines) were predicated. This was especially the case for the efficacy of thrombolysis; management of fever at 37.5 °C and the threshold for treating hyperglycaemia (see Table 1).

Resistance to operationalising the T^3^ clinical protocol element for treating hyperglycaemia at > 10 mmol/L [180 mg/dL] in patients regardless of diabetic state (which was underpinned by the Australian Diabetes Society guideline for the management of all in-patient hyperglycaemia [regardless of diagnosis] to prevent episodes of major hyperglycaemia) [10, 11] was justified by some clinicians on the basis of evidence from the intensive care setting against ‘tight’ glycaemic control (treatment with insulin to keep the glucose between 4.5 and 6.0 mmol/L [81–108 mg/dL]) [20]:

Most senior staff are aware of work done in intensive care units which show that tight glucose control kills people outside of a study environment. So people essentially ignore it (ED Doctor)

The T^3^ trial was great and showed benefit but didn’t prove to me that insulin infusions were the way to go for stroke patients. I say this is because many trials had shown that intensive lowering of glycaemia was harmful (Stroke Unit Senior Nurse)

Similarly, for the protocol element pertaining to thrombolysis (Table 1), there was resistance to implementation despite this element being supported by high certainty research evidence. In addition, medical staff views about aspects of the evidence were reported to influence ED nurses and also had the knock-on effect of acting as a barrier to the implementation of all aspects of the protocols:

The entrenched resistance towards thrombolysis from some ED medical staff has an influence on ED nursing staff. (Stroke Unit Senior Nurse)

There is a culture of misunderstanding about risk versus benefits of thrombolysis. These views have filtered to nursing as well (Emergency Nurse)

The ED doctors were so resistant to thrombolysis that this was a barrier to implementing all protocol elements at our site (Stroke Unit Senior Nurse)

Despite multidisciplinary educational workshops and follow-up education, there were multiple comments that the implementation strategy relied on the evidence ‘selling itself’. One participant noted that while this approach may be effective in a specialised setting such as a stroke unit, settings such as the ED where there are rapidly changing and competing clinical priorities as well as unfamiliarity with the evidence for stroke management, more intensive strategies might be needed.

#### Adherence versus adaptation

All elements of the T^3^ clinical protocols were expected to be implemented with only minor negotiated changes to align with local hospital policies such as administering insulin subcutaneously rather than intravenously. In contrast, examples of an unacceptable adaptation were not treating fever as per protocol or one-off glycaemic measurement rather than 6-hourly monitoring. While some intervention sites reported embedding the protocols into existing ED stroke pathways, others reported that in the dynamic ED environment focused on triage and assessment it was difficult to follow the protocols at all times, hence full adherence to all elements of the protocols was viewed as ‘sustainable in parts’ reflecting that adherence to the frequency required was challenging in the ED. This led to protocol modifications, and partial adoption of the protocols particularly for the fever and hyperglycaemia protocol elements:

It’s not that the protocol was difficult; there’s a difference between the protocol and then people having the time or effort to actually think about and act on it (ED Doctor)

Little bits of the protocol would be dropped off here and there, like part of the temperature and glycaemia monitoring (Senior Emergency Nurse)

Getting temperature within the hour was more challenging than we realised. Particularly if they’re getting thrombolysis within that first period of time

(Stroke Unit Senior Nurse)

Modifications to the protocols also occurred when there were different recommendations in existing hospital documentation that conflicted with the protocols. This ‘resulted in confusion’ (ED doctor) and led to protocol adaptations:

Doctors are unhappy about giving insulin below 11 [mmol/L (198 mg/dL)]. The biggest challenge in protocol adherence was insulin (Emergency nurse)

To address this, required resources and time to develop new documentation requiring that had to be approved by hospital management and this happened rarely with previous practices often continued that conflicted with the protocols.

In summary, beliefs about the supporting evidence and conflict of the protocols with existing hospital policies and pathways led to low uptake of aspects of the protocols and also protocol modifications and adaptations.

### Boundaries of care

#### Defining professional boundaries

There was a strong view expression that ED was not a place for delivery of the ‘specialist’ care needed for patients presenting with stroke. This was seen as being at odds with the role of ED staff as the first-line providers for patients in need of immediate attention. ED clinicians’ views that stroke management is the ‘specialist care’ meant that delivery of the T^3^ clinical protocols was not seen as within the boundaries of ED practice:

Stroke is a specialist disease, therefore the patient shouldn’t be in ED and we should have the stroke unit ringing us and saying ‘How dare you still have my patient, get them up to me’ (ED Doctor)

Many comments highlighted that ED priorities are triage and assessment, and that once a diagnosis of stroke is made, the stroke team should take over as the ED has other urgent priorities:

In a busy ED what we can do acutely is set them up for thrombolysis. To be brutal and this is a slightly unfair comment, but the ED sees its job as almost done once we’ve booked the CT and let the stroke team know. I don’t make a lot of excuses for that because there are a lot of other jobs that we need to do and other staff who can provide stroke care (ED Doctor)

Hence, boundaries were constructed through clinicians limiting their responsibilities to their specialist field and these boundaries impacted upon optimal protocol uptake.

#### Care trajectory

The combination of practical considerations of ED work priorities, beliefs that stroke required a suite of specialist care and the expectation that patients with stroke would be rapidly transferred from ED to a dedicated stroke unit (not borne out by the data [10]) also impeded protocol uptake. Related to professional boundaries, the care trajectory of a patient with acute stroke also influenced adherence to the protocols. Feedback from ED clinicians reflected an assumption that as part of the care trajectory for patients with stroke was timely transfer to the stroke unit (Table 1), then it was not seen as central for ED staff to fully adhere to the protocols for the ongoing monitoring and surveillance elements for fever and hyperglycaemia.

Almost all stroke centres have a very actively engaged stroke team who quickly pull patients out of ED. Therein lies the challenge because you get a loss of interest and engagement from ED (Stroke Unit Senior Nurse)

It would be relatively rare that we’re doing protracted blood sugar or temperature measurements because most of these patients are now going to the ward 60-90 minutes after arrival in ED (ED Doctor)

In addition, once a patient with a stroke was assessed as being stable, then other patients were prioritised.

By definition, ED staff triage everything. So our nursing staff see the patient in the bed next door looks sicker and they're just going to ignore it and that's what they should do, because that's how EDs work (ED doctor)

If a stroke unit bed was unavailable, ED staff expressed a view that this ‘tied up’ ED staff beyond what was perceived to be the appropriate duration of care in ED for patients with stroke: *Not our business … . In general, once they’re admitted and waiting for a bed, unless they acutely deteriorate, we would take very little notice of them* (ED Doctor).

In summary, the participant views were that the monitoring aspects of the T3 protocols to the required degree once the patient was stabilised was seen as disruptive to ED routine practice which has an emphasis on triage and assessment. Professional boundaries and the established care trajectory for patients with stroke were also used as a justification for not fully enacting the protocols until the patient was transferred from ED.

## Discussion

This process evaluation provides important information specific to a contextualised understanding of the uptake of the T3 protocols and contributes knowledge to the broader field of implementation science. The findings are explainable through three themes with sub-themes that have identified, from the perspective of those involved in driving practice change that low medical engagement, beliefs about the supporting evidence held by ED clinicians and perceptions of professional boundaries influenced the implementation and uptake of nurse-led stroke protocols in the ED. These findings fill an important gap in the literature by providing insights into the challenges involved with implementing new and complex interventions for time-critical conditions such as stroke in the ED. The ED is a setting for which there is limited evidence for successful stroke care intervention uptake [21].

The application of the normalisation process theory helped to highlight some important findings. The theme *Readiness to change* showed that achieving medical engagement was difficult. This was despite multidisciplinary education workshops, individual meetings with medical staff to present the evidence and to clarify implementation responsibilities, and ongoing support and education ‘top-ups.’ Lack of sustained medical engagement and a perception that the clinical champions lacked the authority to lead multidisciplinary change were barriers to ‘collective action’ hampered that effective operationalization and integration of the protocols into everyday practice. Other research reports that for multidisciplinary ‘buy-in’ and effective implementation, a critical mass of champions from a range of disciplines is needed to support multidisciplinary practice change and also that compared with other healthcare staff, medical doctors’ involvement in interprofessional educational activities is low [22–24]. Recent research has highlighted the importance of several attributes of clinical champions that could be considered when selecting people to fulfil these roles: influence, ownership, physical presence at the point of change, persuasiveness, grit and participative leadership style [25].

The theme *Fidelity to the protocols* illustrated the impact of entrenched beliefs about the supporting evidence. Staff acceptance and capacity to fully adhere to protocols is central to intervention fidelity [26, 27]. Although minor protocol adaptations (described above) were permissible, full adherence to the ‘minimum intervention elements’ (Table 1) was expected in order to deliver evidence-based care to improve patient outcomes [4, 10]. However, beliefs about aspects of the evidence held by ED medical staff particularly about thrombolysis and hyperglycaemia management appeared to influence other clinical team members and this acted to mitigate against the full implementation of the protocols.

ED staff reported modifying the protocols because they perceived that full implementation of the protocols was time-consuming in relation to other urgent priorities. This was an unforeseen challenge and may indicate a lack of ‘internalisation’ of the protocols [15] by some clinical team members. Using normalisation process theory concepts, this means that while there was an agreement with the aims and expected benefits of protocol implementation, the value and importance of the supporting evidence and its applicability to the ED setting was not fully understood. Furthermore, using a normaliation process theory lens, it may be that a loss of ‘cognitive participation’ may have occurred after the initial multidisciplinary workshops where the supporting evidence and rationale of the protocols were presented [15, 16]. Overcoming clinician ‘mindlines’ and beliefs about the evidence is a vexing challenge in implementation science [28] and in acute stroke management. This is particularly so in relation to the administration of thrombolysis, a key nursing and medical responsibility [29, 30] and a protocol element with clear evidence of effectiveness [4, 5]. As stated by some interviewees, bias against the use of tissue Plasminogen Activator in some sites, also resulted in low acceptance of other elements of the T^3^ clinical protocols. It is of concern that beliefs about aspects of the evidence (including international guidelines) supporting these evidence-based protocols can derail or ‘de-legitimate’ all elements of the protocols as well as inhibit the individual and collective action needed to embed and sustain the use of these protocols [15, 16]. From the perspective of normalisation process theory, an individual appraisal by clinicians of the applicability of the evidence to the ED setting worked against the collective action needed to facilitate use and to normalise the use of the protocols in the ED. Other recent research has shown that ED clinicians express low agreement with the evidence supporting tissue Plasminogen Activator use in acute stroke [29, 30]. It is therefore possible that active and ongoing championing by key medical staff might have helped to reinforce the education given about the evidence that was part of the implementation strategy.

*Boundaries of care* revealed a tension between roles and responsibilities as they related to the different clinical contexts of ED and stroke specialist practice. Boundaries were constructed through clinicians limiting their responsibilities to their specialist field; the routine practicalities of patient transfer between departments and specialties, and through the availability of staff to care for a patient with acute stroke both in the ED and in the stroke unit. These findings are consistent with those from studies highlighting professional role and identity as important factors influencing intervention implementation [31, 32]. There was a strong view that ED was not the place for the delivery of ‘specialist’ stroke care as ED staff are the first-line providers for patients in need of immediate attention. Australian data suggest that ED clinicians primarily attend to patients with fractures, burns and toxic effects of medicinal and non-medicinal substances drug overdoses, with a low volume of stroke presentations [33]. Patients with acute stroke may therefore be regarded by ED clinicians as a lower priority, particularly as they do not present with pain and there is no bedside monitor to measure worsening cerebral infarction. In NPT terms, the assessment and understanding of the value of the T3 protocols for the ED setting (reflexive monitoring) appeared to be negatively impacted by perceptions of professional boundaries and expectations relating to clinical responsibilities at different stages of the patient care trajectory.

Our findings reinforce previous research aimed at implementing new and complex interventions for time-critical conditions in the ED. Results from an evaluation of the implementation of a protocol-based sepsis resuscitation in EDs in the USA highlights issues with performing time-sensitive critical care in the ED with the need for more ED time, resources and nursing staff [34]. A Canadian study evaluating the barriers to implementation of therapeutic hypothermia for cardiac arrest patients in the ED also found that ED staff were reluctant to initiate this time-critical treatment which they considered to be an intensive care unit intervention, with participants describing the ED as ‘treat and go’ [35]. Decreased motivation to adopt changes is not uncommon when staff does not consider the intervention as part of their role [36].

There was also a belief that stroke patients would rapidly transfer out of ED to a stroke unit bed (not borne out by the results of our quantitative analysis [11]). The clinical implications of this were that stroke patients did not receive the full complement of protocol care while waiting for a stroke unit bed. In normalisation process theory terms, a low value was attached to fully enacting the protocols, irrespective of the benefits to patients with stroke [15, 16]. Given that the T^3^ clinical protocols have resulted in significant reductions in death and dependency following a stroke in a previous trial conducted in the acute care setting [4], further exploration is needed about how to ensure patients with stroke receive the full complement of evidence-based management throughout the care trajectory.

The implementation science implications of our study suggest that in the ED setting, if changes are perceived by ED staff to be related to therapeutic interventions beyond those required for a very acute or resuscitation situation then full implementation is likely to be compromised. The findings of a recent study show that the introduction of guidelines into EDs is likely to be unsuccessful if guidelines interfere with ED workflow [9]. ED workflow may therefore compromise the uptake of evidence-based protocol care. Studies have also demonstrated the inability of staff to prioritise new interventions in the ED where these interventions are considered to be outside the role of ED staff which is focused on hyper-acute patients and maintaining patient workflow [31, 36]. Other stroke implementation trials have also experienced similar difficulties in effecting practice change and protocol uptake and cited workforce shortages and the nature of ED workflow as reasons [10, 29, 30]

Despite the potential for the T^3^ clinical protocols to significantly improve patient outcomes, in common with the findings of other studies, successful implementation in hospital systems is not always achieved [36]. The extent to which evidence-based practice is adopted and sustained in clinical service environments is variable and dependent on a range of organisational and contextual factors that may be unrelated to the strength of the evidence or the best intentions of clinicians [36]. Where considerable preparatory and ongoing work has occurred, as in our trial, systematically identifying barriers and facilitators, matching to behaviour change techniques [11–13] and developing site action plans may also influence evidence adoption. Contextual and organisational pressures within which behavioural change occurs may override these implementation efforts and may not be able to be ameliorated within the context of a trial [36]. A challenge of implementation science research is determining effective implementation strategies to address these complex and unforeseen barriers within the constraints of a trial [37].

### Strengths and limitations

The main strength of this process evaluation is the representation from different perspectives of the factors influencing protocol uptake in the emergency department. This study met key recommendations for process evaluation [14, 17], namely that the study was planned a priori to evaluate contextual factors impacting upon the implementation of the T^3^ Trial protocols from the viewpoint of clinical and support staff (Trial State Co-ordinators) involved in the implementation. However, as the process evaluation was conducted at the completion of the T^3^ trial, it is possible that participants may not have recalled some details of the implementation process. The researchers were independent of the T^3^ research team, blind to the main study findings and had the required qualifications to undertake the process evaluation. The use of normalisation process theory to inform the interview schedule, coding and interpretation added depth to the analysis and interpretation of results lending an understanding of factors that supported or constrained normalisation of the intervention into routine practice. We have provided a clear documented audit trail of methods and analysis methods to enable assessment of the rigor of the approach and transferability of findings to other settings. Themes are linked to participant quotes to demonstrate the grounding of the findings in the data.

In common with other studies, there are a few limitations. Data were collected until saturation, but it is possible that more experiences may have been found from additional interviews. However, there was good representation of participants from a range of clinical backgrounds ensuring transferability. Given that the trial included only hospitals with a dedicated stroke team and unit means that these findings may not be generalisable to hospitals with fewer specialist resources for stroke.

## Conclusions

A systematic adaptation of a previously successful nurse-led protocol intervention in stroke units and a theoretically informed implementation intervention could not overcome complex individual and contextual factors. These findings add to the body of knowledge about the implementation of research evidence into clinical practice in the ED setting and will help guide the implementation of future interventions for acute stroke care in the ED. In particular, future implementation interventions in the ED might want to consider strategies to address barriers such as beliefs about the evidence, whether clinical champions are invested with sufficient authority to facilitate collective change in a busy and dynamic clinical setting and the impact of professional boundaries on perceptions of clinical responsibility for executing protocols. Alternative models of care for patients presenting with stroke to the ED should be considered to ensure evidence-based treatment of patients with stroke outside of stroke units.

## Data Availability

The datasets used and analysed during the current study are available from the corresponding author on reasonable request.

## Abbreviations

ASU: Acute stroke unit
ED: Emergency department
NPT: Normalisation process theory
TDF: Theoretical Domains Framework
RCT: Randomised controlled trial
Thrombolysis: Tissue plasminogen activator
